# Comparing Methods for Measurement Error Detection in Serial 24-h Hormonal Data

**DOI:** 10.1177/0748730419850917

**Published:** 2019-06-12

**Authors:** Evie van der Spoel, Jungyeon Choi, Ferdinand Roelfsema, Saskia le Cessie, Diana van Heemst, Olaf M. Dekkers

**Affiliations:** * Section Gerontology and Geriatrics, Department of Internal Medicine, Leiden University Medical Center, Leiden, the Netherlands; † Department of Clinical Epidemiology, Leiden University Medical Center, Leiden, the Netherlands; ‡ Section Endocrinology, Department of Internal Medicine, Leiden University Medical Center, Leiden, the Netherlands; § Department of Biomedical Data Sciences, Leiden University Medical Center, Leiden, the Netherlands

**Keywords:** measurement error, outlier, time series, hormones, automatic outlier detection

## Abstract

Measurement errors commonly occur in 24-h hormonal data and may affect the outcomes of such studies. Measurement errors often appear as outliers in such data sets; however, no well-established method is available for their automatic detection. In this study, we aimed to compare performances of different methods for outlier detection in hormonal serial data. Hormones (glucose, insulin, thyroid-stimulating hormone, cortisol, and growth hormone) were measured in blood sampled every 10 min for 24 h in 38 participants of the Leiden Longevity Study. Four methods for detecting outliers were compared: (1) eyeballing, (2) Tukey’s fences, (3) stepwise approach, and (4) the expectation-maximization (EM) algorithm. Eyeballing detects outliers based on experts’ knowledge, and the stepwise approach incorporates physiological knowledge with a statistical algorithm. Tukey’s fences and the EM algorithm are data-driven methods, using interquartile range and a mathematical algorithm to identify the underlying distribution, respectively. The performance of the methods was evaluated based on the number of outliers detected and the change in statistical outcomes after removing detected outliers. Eyeballing resulted in the lowest number of outliers detected (1.0% of all data points), followed by Tukey’s fences (2.3%), the stepwise approach (2.7%), and the EM algorithm (11.0%). In all methods, the mean hormone levels did not change materially after removing outliers. However, their minima were affected by outlier removal. Although removing outliers affected the correlation between glucose and insulin on the individual level, when averaged over all participants, none of the 4 methods influenced the correlation. Based on our results, the EM algorithm is not recommended given the high number of outliers detected, even where data points are physiologically plausible. Since Tukey’s fences is not suitable for all types of data and eyeballing is time-consuming, we recommend the stepwise approach for outlier detection, which combines physiological knowledge and an automated process.

## Introduction

Many physiological parameters such as hormones or metabolites exhibit rhythmicity. These rhythms are regulated by different systems. The most prominent rhythm is the circadian rhythm, which is induced by the biological clock located in the suprachiasmatic nucleus in the brain. The biological clock not only synchronizes molecular clocks in peripheral cells but also orchestrates many physiological functions including blood pressure, core body temperature, and hormone secretion. An example of a hormone that exhibits a strong circadian rhythmicity is cortisol. The sleep-wake cycle is another form of rhythm, and although similar to the circadian rhythm, it has other effects on hormone secretion than the biological clock. The secretion of growth hormone (GH), for example, is more strongly influenced by sleep than by clock time. Also, external cues, including food intake and physical activity, can influence hormone secretion, such as the secretion of insulin (Oike et al., 2014).

Hormones and metabolites are measured for different purposes, for example, in clinical settings to make a diagnosis or to evaluate the effect of treatment and in research settings to investigate how these parameters change based on interventions or differ between groups. Different cues can elicit changes in hormone secretion, among which are circadian time, nutrient availability and food intake, physical activity, and sleep. Circulating concentrations of many hormones change over time, because these hormones are secreted in a pulsatile fashion and have a relatively short half-life (Spiga et al., 2015). Therefore, to obtain reliable hormonal time-series data, hormones need to be measured in blood that is sampled frequently. For some hormones, such as insulin, the preferred sampling frequency is 2 min because of its short half-life (Porksen et al., 1997). Other hormones, including thyroid-stimulating hormone (TSH), can be measured every 20 min to obtain reliable profiles (Odell et al., 1967; Grossmann et al., 1997). To take into account practical possibilities, half-lives, costs, and ethics, most studies investigating hormone secretion are performed with a sampling frequency of every 10 min during 24 h, as reviewed by Veldhuis et al. (2016) and Roelfsema et al. (2017).

When measuring hormones frequently over time, measurement errors are likely to occur. Measurement errors can be caused by preanalytical experimental variation of various sources, including sample dilution (possibly because of flushing the intravenous line with heparinized saline), or the presence of a blood clot in the sample. Measurement error can influence the outcomes of studies with serial hormonal data. Therefore, it is important to identify measurement errors. Measurement errors are likely to be outliers (Grubbs, 1969), which deviate largely from the overall trend of the data. The challenge is that there is no clear-cut distinction between measurement errors and true biological variation. The starting point to detect measurement errors, however, is by identifying outliers.

No well-established method is yet available to automatically detect measurement errors. Therefore, we aimed to compare 4 methods to detect outliers likely due to measurement errors in 24-h hormonal data: eyeballing (relying on experts’ opinions), Tukey’s fences (identifying outliers based on interquartile ranges), stepwise approach (identifying outliers based on standard deviations), and the expectation-maximization (EM) algorithm (using a mathematical algorithm based on disentangling the 2 different distributions of outliers and nonoutliers). Furthermore, we studied the influence of removing the detected outliers on the assessment of statistical features of 24-h hormonal data such as mean, minimum, maximum, and cross-correlation.

For this study, we used data on the pituitary hormone GH, adrenocorticotropic hormone (ACTH), TSH, the adrenal hormone cortisol, as well as data on the metabolic signals insulin and glucose, all of which were measured every 10 min for 24 h in serum from 38 participants of the Switchbox Leiden Study (Jansen et al., 2015).

## Methods

### Data Collection

#### Study population

The Leiden Longevity Study comprises 421 families with at least 2 long-lived Caucasian siblings fulfilling the age criteria (men ≥89 years and women ≥91 years) without selection based on health or demographics (Westendorp et al., 2009). In the current study, the Switchbox Leiden Study, we included 20 offspring of long-lived families from the Leiden Longevity Study together with 18 partners of the offspring as environmental and age-matched controls. The primary aim of the Switchbox Leiden Study was to compare the levels and dynamics of hormones and metabolites and their interplay between offspring of long-lived families and controls. Inclusion and exclusion criteria were described previously in detail (Jansen et al., 2015). Participants were middle-aged (52-76 years) and had a stable body mass index between 18 and 34 kg/m^2^. The Switchbox Leiden Study was approved by the Medical Ethical Committee of the Leiden University Medical Centre and was performed according to the Helsinki Declaration. All participants gave written informed consent for participation.

#### 24-h blood sampling

The 24-h blood-sampling procedure started with placing a catheter in a vein of the forearm of the nondominant hand, and blood withdrawal started around 0900 h (Akintola et al., 2015). Samples of 2 mL serum and 1.2 mL EDTA plasma were withdrawn every 10 min. To prevent blood clotting, heparinized saline (0.9% NaCl) was continuously infused via an infusion pump at a rate of 20 mL/h. Before each blood withdrawal, 5 mL of saline/heparin mixed with blood was collected (without disconnecting the syringe from the blood withdrawal system to prevent contamination of heparin/saline in the blood samples). After blood withdrawal, this 5 mL was flushed back into the subject, to reduce the total amount of blood that would be withdrawn. Participants received standardized feeding consisting of 600 kcal Nutridrink (Nutricia Advanced Medical Nutrition, Zoetermeer, the Netherlands) at 3 fixed times during the day. Participants were not allowed to sleep during the day, and except for lavatory use, no physical activity was allowed during the study period. Lights were switched off for approximately 9 h (circa between 2300 h and 0800 h) to allow the participants to sleep.

#### Assays

All laboratory assays were performed with fully automated equipment and diagnostics from Roche Diagnostics (Almere, the Netherlands) at the Department of Clinical Chemistry and Laboratory Medicine of the Leiden University Medical Centre in the Netherlands.

TSH, cortisol, insulin, and glucose were measured in the same serum tube. GH was also measured in the same serum tube but after 1 additional freeze-thaw cycle. TSH and cortisol were measured by electrochemoluminescence immunoassay using a Modular E170 Immunoanalyzer from Roche Diagnostics. For TSH, the overall interassay coefficients of variation (CV) ranged between 1.41% and 4.16% in our study, and the overall CV of cortisol ranged between 2.4% and 5.1%. Human GH with a molecular mass of 22 kDa and insulin were measured using an IMMULITE 2000 Xpi Immunoassay system (Siemens Healthcare Diagnostics). The interassay CV of GH ranged between 5.4% at 5.43 mU L^–1^ and 7.2% at 25.0 mU L^–1^, and the overall CV of insulin ranged between 3.19% and 7.69%. Glucose was measured using the Hitachi Modular P800 from Roche Diagnostics, and the overall interassay CV of glucose ranged between 0.90% and 7.44%. If a measurement was below the detection limit, half of the lower detection limit was taken as a result.

Although ACTH was also measured, we did not use these data in our mathematical models because this hormone was measured in EDTA plasma (i.e., in a different tube from the other hormones). However, for the aim of eyeballing, we did use ACTH data, as these data were instrumental for inspecting physiologically abnormal points in the cortisol data.

### Physiological Considerations

Since hormones are secreted in a pulsatile manner, a sudden increase is more likely to occur than a sudden decrease. Also, a glucose level <2.8 mmol/L does not occur in healthy persons without an accompanying strong stress response (cortisol and GH pulses). ACTH stimulates the secretion of cortisol; therefore, cortisol should show a pulse following an (extreme) increase in ACTH. If an outlier is caused by sample dilution, then all hormones measured in that sample should be lower than expected. These physiological considerations could be taken into account in measurement error detection.

### Methods of Detecting Outliers

**Figure 1.**
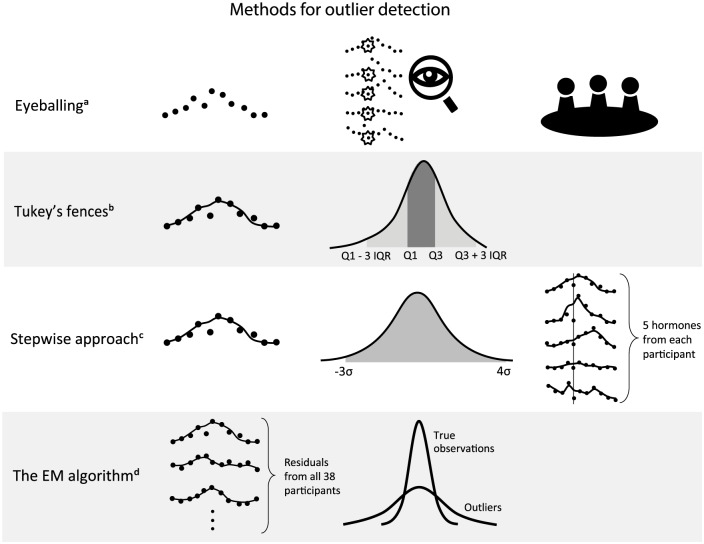
(a) Eyeballing detects outliers without fitting smooth curves. By visual inspection, individual experts detect outliers by taking into account that some hormones were measured in a same sample. Afterward, a consensus meeting is held, and the experts discuss all data points with conflicting detection results. (b) Tukey’s fences starts with fitting a moving average curve to per-person per-hormone data and taking residuals of all data points. Then the interquartile range (IQR = Q3–Q1) of the residuals is calculated. The data points lying outside the range between Q1 − 31QR and Q3 + 3IQR are detected as outliers. (c) The stepwise approach fits the moving average curve to per-person per-hormone data, and standardized residuals of all data points are calculated (step 1). The data points lying outside the range between −3 and 4 standard deviations are detected as outliers (step 2). Then, the residuals of 5 hormones measured at the same time points are summed. When the sum of the residuals is smaller than −8, the data points are detected as outliers (step 3). Afterward, steps 1 and 3 are repeated (step 4). (d) The expectation-maximization (EM) algorithm first fits a smoothing curve to per-person per-hormone data, and the residuals are calculated. Then, all the residuals of a hormone from all 38 participants are put in the EM algorithm. The algorithm then identifies 2 distinguishable distributions and yields the probability of each data point to be an outlier.

In the following section, we will discuss 4 methods for outlier detection: (1) eyeballing, (2) Tukey’s fences, (3) stepwise approach, and (4) the EM algorithm. The procedures of these methods are visualized in Figure 1.

#### Eyeballing

Eyeballing was based on visual inspection of a graphical display of individual hormone profiles from all 38 patients. This was performed by 4 reviewers with expert knowledge in endocrinology (E.v.d.S., F.R., O.M.D., and D.v.H.). Hard copies of the 24-h trajectories of all hormones measured per participant were provided. Three reviewers examined all 38 participants’ hormone profiles, and 1 reviewer checked half of the participants. Information about which hormones were measured in the same tube was given verbally. Reviewers were also explicitly told that dilution of the sample may have led to measurement errors in all hormones measured from the same tube. After reviewing the data separately, a consensus meeting was held to reach agreement on data points of which only 1 (out of 3 or 4) or 2 out of 4 reviewers had marked as an outlier.

#### Tukey’s fences

For this algorithmic approach of outlier detection, we made the following assumptions: (1) a hormone trajectory of an individual person follows a smooth general trend over 24 h while measurement errors may deviate clearly from the trend, and (2) hormone levels cannot abruptly decrease within 10 min. If a measurement is vastly distant from the adjacent measurements before and after, that measurement is likely to be a measurement error. Thus, by fitting a smooth curve to the data points and measuring the distance between the curve and each measurement, the algorithm can detect outliers expected to be measurement errors.

Tukey’s fences is a nonparametric method developed to detect observations out of the normal range by using interquartile ranges (Tukey, 1977), and it is often used for detecting outliers in various fields (Muraleedharan et al., 2016; Pham and Eggleston, 2016; Luo et al., 2018; O’Brien et al., 2018). Before performing Tukey’s fences, normality of the data was checked before fitting the curve. The distributions of insulin and GH data were highly skewed; therefore, these data were log transformed prior to applying the algorithm. Afterward, Tukey’s fences was implemented using the following 2 steps:

1. Hormone data were smoothed over time by fitting moving average curves for every hormone per person separately. Moving average is a method commonly applied for smoothing time-series data (Montgomery et al., 2015). The moving average with window size *n* (with *n* being an odd number) at a certain time point is the average of the current, the −½(*n* − 1) previous, and ½(*n* − 1) subsequent measurements in time. In our analyses, moving averages were calculated using a window of 5 points. Residuals were calculated for all data points. We defined a residual as the vertical distance between an original data point and a fitted moving average curve.
2. In the second step, the interquartile range (IQR) of the residuals, the distance between the first quartile and the third quartile (*Q*_1_ − *Q*_3_), and the median (*Q*_2_) were identified. The ranges between *Q*_2_ − *k*(*Q*_3_ − *Q*_1_) and *Q*_2_ + *k*(*Q*_3_ − *Q*_1_) are referred to as fences. The data points that are below the lower fence or above the higher fence are identified as outliers. The value *k* determines the width of the fences. The larger the value of *k*, the lower the number of outliers that will be detected. In our analyses, we set *k* = 3, which, according to the literature, implies that the data point is “far out” (Tukey, 1977). To use the method as it is originally suggested and commonly being applied, we did not adjust the value of *k* = 3 (Horn et al., 1988; Hung and Yang, 2006; Kimenai et al., 2016).

#### Stepwise approach

The stepwise approach is an automatic detection process based on an algorithm that incorporates physiological knowledge and statistical methods and comprises 4 steps, as described below. Our aim was to detect potential outliers within a 24-h hormone trajectory in several steps. As in Tukey’s fences, the insulin and GH data were log transformed.

1. *Step 1: Fitting smoothed curves.* Likewise to Tukey’s fences, a moving average curve is fitted to each participant’s 24-h hormone data using a window of 5 points. By computing the distance between each data point and the fitted curve, residuals are acquired. The residuals are standardized to have a mean of 0 and a standard deviation of 1.
2. *Step 2: Detecting outliers within a 24-h hormone trajectory.* Data points with standardized residuals smaller than −3 or larger than 4 are detected as outliers. The cutoff of 3 standard deviations is a commonly applied empirical rule for detecting outliers in normal distributed data. However, asymmetrical cutoffs are chosen to be more liberal for the upper boundary, as hormones are secreted in a pulsatile fashion, which makes rapid increases in hormone level biologically more plausible than rapid decreases, since clearance of the hormone will occur more slowly. Note that this cutoff boundary is wider than the width of Tukey’s fences with *k* = 3. Furthermore, data points at which glucose <2.8 mmol/L were detected as outliers, as discussed in the Physiological Considerations section.
3. *Step 3: Detecting outliers across hormones.* The standardized residuals of all hormones measured in the same serum tube are added for each participant. If the sum of the standardized residuals is lower than −8, all data points measured in that tube are detected as outliers. This means that the residuals of the 5 hormones are on average below the fifth percentile of standard normal distribution (1.64 standard deviation). This step allows for the detection of measurement errors due to dilution of the samples. The underlying assumption is that when samples were diluted, levels of the hormones measured in the same sample are all likely to be lower at the same time point. In this step, we aim to detect these types of measurement errors that occur across the hormones.
4. *Step 4: Repeat step 1 and step 3.* After all outliers detected so far are removed, a new moving average curve is fitted, and steps 1 and 3 are repeated once. If already detected outliers are removed, the newly fitted curves will be flatter than the fitted curve from the original data, which will allow the detection of potential outliers that were missed in the previous steps.

#### The EM algorithm

Another approach is to estimate the probability for a data point to reflect measurement error, rather than using a dichotomous division. This starts with assuming 2 distinguishable data distributions: true measurement variation and background noise due to measurement errors. Based on this assumption, we expect the residuals of the true measurements to be normally distributed with standard deviations close to 0, while those of the erroneous measurements would be normally distributed with a larger standard deviation. The EM algorithm is a method that can be used to identify these 2 distinguishable distributions. The algorithm estimates model parameters when data are incomplete or when the model depends on a latent variable—a variable that is not directly observed but can be inferred by other observed variables (Dempster et al., 1977)—and the method was suggested for detecting outliers (Aitkin and Wilson, 1980). The EM algorithm was applied in R version 3.5.1, using the normalmixEM function of the package mixtools (Benaglia et al., 2009). In our situation, the latent variable of interest would be whether a data point is a true measurement or a measurement error. Further technical details about the EM algorithm can be found in the Supplementary Material, Appendix 1.

The EM algorithm has the advantage that detected outliers do not have to be removed. Instead, the probabilities can later be used as weights for estimating outcomes, such as mean hormone levels or cross-correlations.

The outlier detection method using the EM algorithm followed the steps below. Again, insulin and GH data were log transformed.

1. As in Tukey’s fences and stepwise approach, a moving average curve per 24-h hormone profile for each individual participant was fitted. Afterward, residuals were calculated and standardized for each data point.
2. The EM algorithm was applied for each hormone with residuals of all participants together taken into account in one model.

### Comparing Methods on Statistical Outcomes

Since we do not know with certainty which data points reflect measurement errors, it is not possible to ascertain which of the 4 methods performed best. Therefore, we compared the number of outliers detected that were counted per time point and in total data points. In addition, the overlap in detected outliers between the 4 methods was visually presented with Venn diagrams (Larsson, 2018). We chose these parameters since these descriptive statistics give a transparent description of the data and will give an insight into what impact removing outliers has on general measures.

Furthermore, we analyzed statistical outcomes of 24-h hormonal data before and after removing the outliers as detected by the 4 different methods. In this way, we could investigate whether removing outliers influenced the statistical outcome and how different methods may do so differently. Therefore, the 24-h means, median, minima, and maxima of the 5 hormones were assessed, which provides a transparent description of the data and insights on how removing outliers affects general measures. Another relevant analysis is cross-correlation between 2 hormones. Cross-correlation estimates the temporal relationship between 2 hormonal concentrations. It is a common analysis performed with data of 2 simultaneously measured hormonal time series (Vis et al., 2014). Therefore, it could be of interest for researchers to know to which extent measurement error would affect the estimates, especially since this method might be sensitive to the presence of outliers that co-occur in different time series data, for example, due to the dilution of a sample. Two relevant outcome measures are the strongest correlation coefficient (the maximal correlation) and the correlation coefficient at lag time 0. For the purpose of this article, we performed cross-correlation on concentrations of glucose and insulin, which are expected to display strong cross-correlation (Feneberg et al., 1999). When estimating the mean and cross-correlations after outlier removal by the EM algorithm, the weighted mean and weighed correlation are calculated, with the weight equal to the probability of each data point to be an outlier. All statistical analyses were performed using the software program R, version 3.5.1.

## Results

For each of the 38 participants, blood samples were collected at 144 time points over 24 h, with 5 hormones being measured in the same serum tube. After discarding missing data, the total number of data points was 21,467. We counted detected outliers per time point and in total data points. If counted per time point, at least 1 outlier was detected in a time point among all hormones assayed in serum (i.e., glucose, insulin, TSH, cortisol, and GH). In case of a complete series, a single participant has 144 time points for each hormone. If counted in total data points, every data point is counted individually. In the case of a complete data set, 1 participant has in total 720 data points, that is, 144 time points times 5 hormones.

### Number of Detected Outliers

Table 1 summarizes the mean percentage of outliers detected per time points and in total data points. The results are averaged across 38 participants. Since the EM algorithm yields continuous probability as its outcome, we defined a data point of which its probability to be an outlier is higher than 0.9 as an outlier. For the percentage of detected outliers, we observed some differences between the 4 methods. Eyeballing resulted in the smallest percentage of detected outliers both per time point (mean = 1.7%) and for total data points (1.0%), followed by the stepwise approach (per time points: 5.1%, total data points: 2.7%). Tukey’s fences yielded more outliers per time point (9.3%) but a similar percentage in total data points (2.3%). The EM algorithm method yielded the largest percentage of outliers (per time points: 40.3%, total data points: 11.0%).

**Table 1.** Percentage of time points with at least 1 detected outlier among the hormones measured and the percentage of total data points detected as outliers among the same set of hormones.^a^

	Mean (SD), *N* = 38	
	Time Points Detected to Contain an Outlier (%)	Total Data Points Detected to be Outliers (%)
Eyeballing	1.7 (2.1)	1.0 (1.4)
Tukey’s fences	9.3 (5.6)	2.3 (1.4)
Stepwise approach	5.1 (1.5)	2.7 (1.5)
EM algorithm^b^	40.3 (7.7)	11.0 (2.8)

a. Mean and standard deviation in the 38 participants are given.

b. For the EM algorithm results, the measurement points at which the probability to be an outlier was >0.9 were counted.

**Figure 2.**
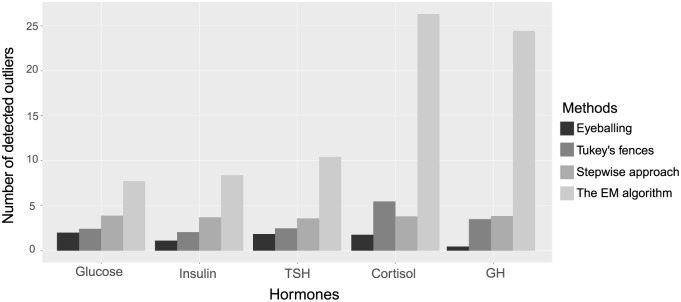
Mean number of data points detected per hormone per method across all participants.

In Figure 2, the numbers of detected outliers for each hormone averaged over all participants are presented. The EM algorithm detected more outliers compared to the other methods, especially in cortisol and GH. Eyeballing, Tukey’s fences and the stepwise approach detected a similar number of outliers across the different hormones.

### Overlap in Detected Outliers

**Figure 3.**
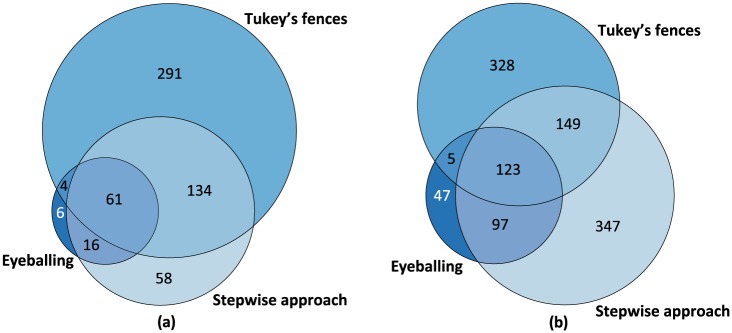
Venn diagrams visualizing the number of measurement errors detected by each method (eyeballing, stepwise approach, and Tukey’s fences) and their overlap counted in total time points (a) and in all data points (b). The overlap with the expectation-maximization algorithm is not presented here for the reasons mentioned in the Results section.

Figure 3 displays Venn diagrams presenting the number of outliers detected by eyeballing, stepwise approach, and Tukey’s fences and their overlap. We did not include the results of the EM algorithm in the Venn diagrams for 2 reasons: (1) the EM algorithm detected an implausibly large number of outliers (per time point = 1590 and in total data points = 2728) and (2) 3 sets of data is the maximum to draw a proportional Venn diagram in 2-dimensional space. Figure 3a presents the number of outliers per time point, and Figure 3b presents that of the total data points. In Figure 3a, most of the outliers detected by eyeballing were also detected by the other 2 methods, while the overlap was larger with the stepwise approach. In Figure 3b, the overlap between eyeballing and the stepwise approach was again larger than the overlap between eyeballing and Tukey’s fences. Here, the stepwise approach and Tukey’s fences detected a similar number of outliers. However, the overlap was relatively small, which indicates that they detected different data points. Eyeballing detected 47 total data points, which were not detected by stepwise approach or Tukey’s fences. Among outliers per time point detected by eyeballing, stepwise approach, and Tukey’s fences, 95.8% overlapped with the outliers detected by the EM algorithm (data not shown). In addition, 70.1% of the total data points detected by the 3 methods overlapped with the outliers detected by the EM algorithm (data not shown).

### Representative 24-h Hormone Figures Presented with Detected Outliers

**Figure 4.**
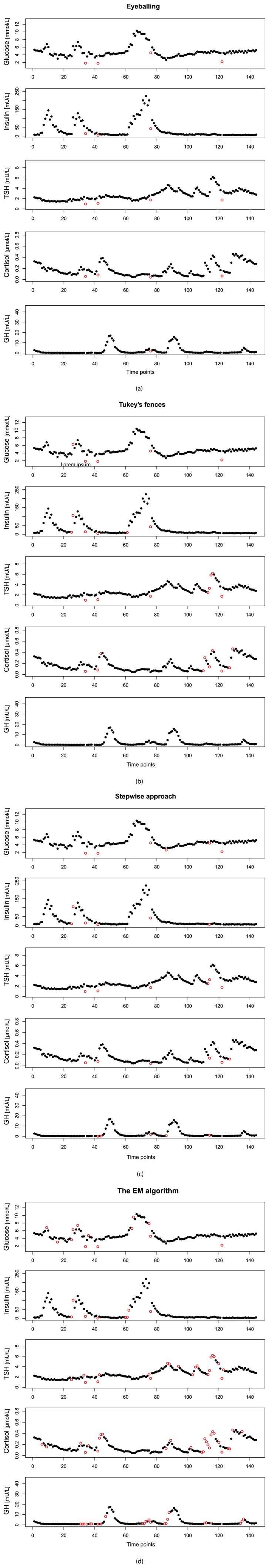
(a) The results of outlier detection by eyeballing in glucose, insulin, thyroid-stimulating hormone (TSH), cortisol, and growth hormone of participant 19. Hollow data points indicate detected outliers (b) The results of outlier detection by Tukey’s fences Hollow data points indicate detected outliers (c) The results of outlier detection by stepwise approach Hollow data points indicate detected outliers Hollow data points indicate detected outliers (d) The results of outlier detection by the expectation-maximization algorithm. Hollow data points indicate the probability of the data point to be an outlier is higher than 0.9.

Figures 4a–d display the detected outliers in glucose, insulin, TSH, cortisol, and GH for eyeballing, Tukey’s fences, stepwise approach, and the EM algorithm, respectively, in one representative participant. By eyeballing (Fig. 4a), 4 data points are detected as outliers in glucose, TSH, and cortisol, and these 4 outliers are all in the same time points. Of these 4 time points, outliers in insulin were detected in 3 time points and in GH in 1 time point. Tukey’s fences (Fig. 4b) detected the same outliers for glucose, insulin, TSH, and cortisol but detected several more than eyeballing. In both TSH and cortisol between time points 110 to 130, several points that are biologically unlikely to be measurement errors were detected. No outliers were detected in GH. The stepwise approach (Fig. 4c) identified the same outliers as eyeballing; however, several extra points were detected as well. Here in several time points (42nd, 76th, and 114th), outliers were detected in all hormones, which is a result of step 3 of the stepwise approach. The EM algorithm (Fig. 4d; note that the points are marked only if the probability of being an outlier is higher than 0.9) resulted in many detected outliers in the pulses that are unlikely to be outliers. Remarkably, in GH, data points close to detection limits were detected as outliers.

### Effects of Removing Outliers on Statistical Outcomes

#### Descriptive statistics: 24-h mean, median, minimum, and maximum

The mean, median, minimum, and maximum values for every hormone were calculated over time before and after removing outliers detected by the 4 methods. This is shown in Table 2. Mean and median values did not change substantially after outlier removal. Minimum values changed for glucose and TSH after removing outliers by all 4 methods, while for insulin, the value did not change much after eyeballing. The EM algorithm had the largest influence on maximum values in all hormones.

**Table 2.** Mean, median, minimum, and maximum values for glucose, insulin, thyroid-stimulating hormone (TSH), cortisol, and growth hormone (GH) in 24 h, before (raw data) and after outlier removal (eyeballing, Tukey’s fences, stepwise approach, and the expectation-maximization (EM) algorithm).

	Mean (SD), *N* = 38											
	Glucose (mmol/L)				Insulin (mU/L)				TSH (mU/L)			
	Mean	Median	Min	Max	Mean	Median	Min	Max	Mean	Median	Min	Max
Raw data	5.09 (0.36)	4.80 (0.39)	2.76 (0.70)	9.51 (1.52)	19.90 (10.11)	9.66 (5.51)	2.76 (2.41)	91.61 (54.41)	2.02 (1.05)	1.92 (1.01)	1.01 (0.61)	3.57 (1.89)
Eyeballing	5.11 (0.36)	4.81 (0.39)	3.16 (0.53)	9.48 (1.47)	19.96 (10.14)	9.66 (5.52)	2.80 (2.46)	91.61 (54.41)	2.03 (1.05)	1.93 (1.02)	1.21 (0.67)	3.57 (1.89)
Tukey’s fences	5.07 (0.37)	4.80 (0.39)	3.04 (0.62)	9.21 (1.42)	19.96 (10.16)	9.69 (5.52)	3.39 (2.39)	91.34 (54.69)	2.02 (1.04)	1.93 (1.02)	1.19 (0.63)	3.49 (1.82)
Stepwise approach	5.12 (0.36)	4.80 (0.39)	3.29 (0.41)	9.40 (1.48)	20.47 (10.43)	10.21 (5.86)	3.54 (2.31)	91.03 (54.26)	2.02 (1.05)	1.92 (1.01)	1.21 (0.64)	3.50 (1.81)
EM algorithm^a^	5.00 (0.37)	4.77 (0.40)	3.14 (0.58)	9.08 (1.47)	20.05 (10.35)	10.16 (5.97)	3.74 (2.44)	87.65 (49.73)	1.98 (1.01)	1.90 (1.00)	1.24 (0.73)	3.33 (1.64)
	Cortisol (µmol/L)				GH (mU/L)							
	Mean	Median	Min	Max	Mean	Median	Min	Max				
Raw data	0.21 (0.05)	0.18 (0.05)	0.05 (0.03)	0.57 (0.09)	2.49 (1.51)	0.95 (0.94)	0.16 (0.22)	20.63 (10.31)				
Eyeballing	0.21 (0.05)	0.18 (0.05)	0.05 (0.03)	0.57 (0.09)	2.48 (1.58)	0.95 (0.94)	0.16 (0.22)	20.63 (10.31)				
Tukey’s fences	0.20 (0.05)	0.18 (0.05)	0.05 (0.03)	0.55 (0.09)	2.47 (1.55)	0.95 (0.94)	0.17 (0.22)	20.27 (10.67)				
Stepwise approach	0.21 (0.05)	0.18 (0.05)	0.05 (0.03)	0.56 (0.09)	2.51 (1.54)	0.96 (0.95)	0.17 (0.22)	20.27 (10.59)				
EM algorithm^a^	0.18 (0.04)	0.16 (0.05)	0.05 (0.03)	0.50 (0.08)	2.24 (1.48)	0.94 (1.02)	0.18 (0.22)	18.90 (11.13)				

Mean and standard deviation in the 38 participants are given.

a. For the EM algorithm results, weighted mean and standard deviation are used.

#### Cross-correlation of glucose and insulin

**Figure 5.**
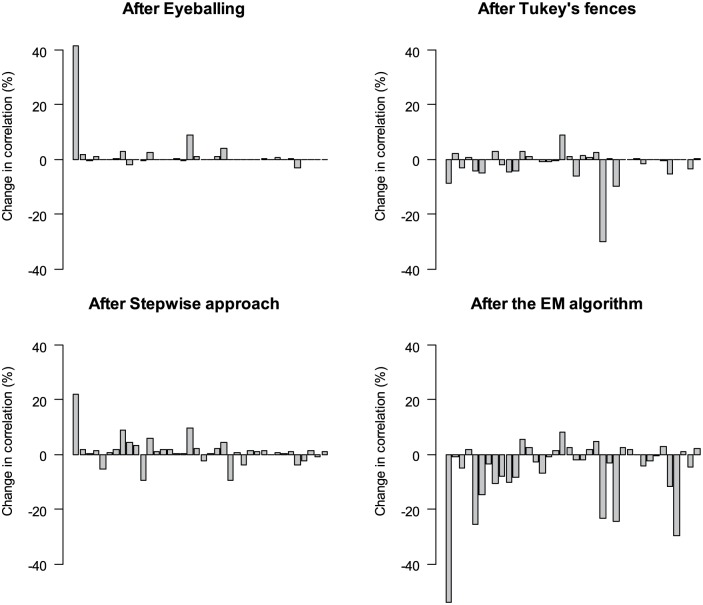
Change in correlation at lag time 0 (%) after removal of measurement errors detected by the 4 methods: eyeballing, Tukey’s fences, stepwise approach, and the expectation-maximization algorithm. Each bar represents an individual participant.

In Table 3, cross-correlations between glucose and insulin are presented before and after removing outliers. Overall, removing outliers did not have a major influence on the cross-correlation of glucose and insulin nor on the lag time at the maximum cross-correlation. Figure 5 shows the individual changes in correlation at lag time 0. In Figure 5, we observe large differences between participants. Especially, the first participant shows a big change in correlation after removing outliers by all methods. Overall, the changes after eyeballing, Tukey’s fences, and stepwise approach were mostly small, and the changes were not toward one direction dominantly. However, after removing outliers detected by the EM algorithm, cross-correlation decreased in most cases.

**Table 3.** Cross-correlations between glucose and insulin.

	Mean (SD), *N* = 38		
	Correlation at Lag Time 0	Maximum Cross-correlation	Lag Time at Maximum Cross-correlation (min)
Raw data	0.74 (0.12)	0.74 (0.12)	−4.7 (7.3)
Eyeballing	0.74 (0.11)	0.75 (0.12)	−5.3 (7.6)
Tukey’s fences	0.73 (0.14)	0.74 (0.14)	−6.3 (8.2)
Stepwise approach	0.74 (0.12)	0.75 (0.12)	−5.0 (8.0)
EM algorithm^a^	0.71 (0.12)	0.73 (0.17)	−9.5 (9.8)

Mean and standard deviation across 38 participants.

a. For the expectation-maximization (EM) algorithm results, weighted correlation is used.

## Discussion

In this study, we aimed to evaluate and compare different methods to detect outliers in 24-h hormonal data since no specific methods were routinely available for this purpose. We assumed that measurement errors will deviate largely from physiological curves of hormones. By identifying outliers in the data, we therefore expected to detect likely measurement errors. The main outcomes of this study were that human judgment (eyeballing) defined fewer data points as outliers than the 3 automatic approaches. Among the automatic approaches, the data-driven methods (Tukey’s fences and the EM algorithm) were prone to detect more outliers likely to be true measurements than the method involving subject-specific knowledge (stepwise approach). The mean, minima, and maxima of the hormones did not change much after removing outliers. However, the minima of glucose and TSH did change, and the EM algorithm had a large influence on maximum values in all hormones. The effect of removing outliers on the correlation between glucose and insulin can be large within an individual but had no major impact on a group level.

A relatively low number of outliers were detected by eyeballing. This may be an advantage of this method as only truly deviating points will be discarded in the analysis. Another advantage of eyeballing is that the data points detected as outliers are based on physiological arguments and not data driven. This allows eyeballing to detect (1) a sequence of data points that was physiologically implausible to display the same pattern in several hormones and (2) outliers at the beginning or end of a time series. These types of outliers cannot be detected by fitting smoothing curves, which explains the 47 data points that were exclusively detected by eyeballing and not by stepwise approach or Tukey’s fences. However, a disadvantage of eyeballing is that it is time-consuming and depends on the individual reviewer’s background knowledge and subjective decision. If the number of reviewers is large enough and a consensus meeting is held, the precision may increase; however, the amount of time to reach a unanimous decision would take longer. Also, eyeballing is a one-off process that cannot be generalized to other settings.

Although Tukey’s fences is advocated as a nonparametric approach, the method did not perform well in our case when applied with moving median curves instead of moving average curves. Especially when the hormone profile is mostly flat with sudden pulses, such as GH, Tukey’s fences with moving median curves detected a biologically implausible number of outliers (54.6% of the total data points). Therefore, when using Tukey’s fences to detect outliers, we suggest researchers be aware of the type of their data and smoothing methods.

We introduced the stepwise approach as a new method to detect measurement errors in 24-h hormonal data. The advantage of the stepwise approach is that by using the standardized residuals, it facilitates detection of measurement errors caused by dilution, which may not have been identified by looking only into individual hormones. In addition, it is expected to be a more objective method than eyeballing, as it explicitly incorporates the information from multiple hormones and applies the same cutoff values of standard deviations to every hormone. Furthermore, it is less time-consuming than eyeballing and can be applied relatively easily to different hormonal data sets. Compared with Tukey’s fences, the stepwise approach has more flexibility to incorporate physiological knowledge, such as adopting an asymmetrical cutoff or removing glucose measurements lower than 2.8 mmol/L. However, the performance of the method may depend on parameters such as a time window for moving average or cutoff points of standard deviations. These parameters still require decisions and need to be chosen with care; the decisions should also be clearly reported. Another disadvantage of the stepwise approach, which also applies for eyeballing and Tukey’s fences, is that we discard data according to a dichotomous division. Whether a data point is an outlier or not is often dependent on the degree of belief instead of a clear dichotomous distinction. Furthermore, this dichotomous distinction reduces the statistical power in further analyses.

The strength of the EM algorithm is that, instead of the dichotomous distinctions, it gives probabilities of each point to be an outlier. Therefore, we acquire extra information that can be incorporated in further analysis such as for probability weighting. In addition, the EM algorithm requires less prior knowledge compared with the previously discussed methods. However, a critical disadvantage of the EM algorithm is that we cannot ensure whether the 2 identified distributions are actually distinguishing outliers and nonoutliers. In our data set, it was not plausible for the detected points to be detected as outliers from a physiological perspective.

The performances of Tukey’s fences, stepwise approach, and the EM algorithm could depend on which smoothing technique is applied. Moving average, which was used in the study, does not require extensive modeling and is able to capture local fluctuations of hormone concentration. However, it may smooth out the transient increase of hormone concentration and lead to the detection of true measurements as outliers. The stepwise approach takes this shortcoming of moving average into account by setting different cutoff values for positive and negative residuals. There are, however, more advanced model-based smoothing techniques, such as deconvolution analysis, which takes underlying dynamics of hormone secretions into account (Brown et al., 2001; Faghih et al., 2014). These methods were not considered in this study as our aim was to compare outlier detection methods that could be easily adopted by applied researchers in a preanalysis phase.

To test the efficacy of the outlier detection methods, we simulated 24-h hormonal data and measurement errors as comparable as possible to real data. The advantage of the simulation study is that we know which data points are true measurement errors. We compared the performance of the stepwise approach, Tukey’s fences, and the EM algorithm. The simulation description and the results are attached as an appendix (see Supplementary Material, Appendix 2). The EM algorithm resulted in a high percentage of true measurements wrongly detected as errors, especially when a simulated hormone has a higher variation during the day than during the night. Most methods yielded relatively low percentages of true errors detected. This could be due to the fact that some simulated errors are close to fitted curves, while the methods we compared are based on detecting errors that deviate from the curves. For detecting dilution errors, the stepwise approach performed better than the other methods. This is because the stepwise approach could detect dilution errors that did not deviate much from the curves by taking the sum of the residuals from all hormones.

In this study, the effect of removing outliers on the cross-correlation between glucose and insulin had no major impact at a group level. Note that these results may not be generalized to other statistical outcomes, such as deconvolution analysis and approximate entropy analysis, which are also common analyses for 24-h hormonal data. Furthermore, glucose and insulin are strongly cross-correlated; however, when 2 hormones are less strongly correlated, the impact of removing outliers may be higher.

## Conclusions

Based on our results, we generally recommend the methods that incorporate physiological knowledge over the data-driven methods. The EM algorithm is not recommended for outlier detection in 24-h hormonal data, since the method seems to falsely distinguish true biological variations due to circadian factors, such as meal response or day-night differences, as outliers. Tukey’s fences, the other data-driven method, is not recommended in 24-h hormonal data. Since no statistical assumptions have to be made and fewer data points will be removed, eyeballing could be a good method for detecting outliers. However, since it is time-consuming (depending on the number of participants studied), it might not always be practical. The strengths and limitations of each method are presented in Table 4.

**Table 4.** Characteristics of 4 outlier detection methods.

	Eyeballing	Tukey’s Fences	Stepwise Approach	The Expectation-maximization Algorithm
Underlying assumptions	• Researchers’ expert knowledge is reliable	• Hormones follows a smooth trajectory over 24 hours		• Two distinguishable distributions (outliers/ non-outliers)
Efficiency and generalizability of the method	• Relatively time-consuming process • Different experts’ knowledge is required for different types of data	• Although it needs several adjustments for different types of time series (e.g., parameters for smoothing curves), the processes can be easily applied to different settings		
Strength and limitations	• Explicit knowledge and clear physiological reasoning behind the detection process • Disagreement between experts may happen	• The method is highly affected by smoothing techniques and the type of data, especially when the hormone levels are mostly constant over time	• Measurement error both within a hormone and within a sample can be detected	• Yields a probability • Need a large sample to be able to distinguish two distributions

In conclusion, we recommend the stepwise approach for detecting outliers in serial 24-h hormonal data, since this method combines both physiological knowledge and an automated process. However, decisions such as which standard deviation cutoffs should be applied or which hormones can be used together in the method should be supported by solid physiological knowledge. The stepwise approach is especially suitable for data of several hormone measurements from the same tube and when dilution is a possible cause of measurement errors. In this case, the outlier detection process can be improved by taking, alongside the hormonal measurements, a reference measurement whose concentration is stable over the day, such as creatinine or urea.

Although the methods showed different performances in outlier detection, this had little impact on the statistical outcomes. Overall, 24-h means and cross-correlations did not change materially, but on an individual basis, correlations might change. The influence of outliers might depend on the study’s sample size and outcome of interest. We recommend researchers be aware of the potential influence of measurement errors in their study, consciously decide which method to choose for outlier detection, and determine whether it is necessary to remove outliers at all.

## Supplemental Material

Appendix_1_Spoel_and_Choi – Supplemental material for Comparing Methods for Measurement Error Detection in Serial 24-h Hormonal Data Click here for additional data file. Supplemental material, Appendix_1_Spoel_and_Choi for Comparing Methods for Measurement Error Detection in Serial 24-h Hormonal Data by Evie van der Spoel, Jungyeon Choi, Ferdinand Roelfsema, Saskia le Cessie, Diana van Heemst and Olaf M. Dekkers in Journal of Biological Rhythms

## Supplemental Material

Appendix_2_final_Spoel__Choi_1 – Supplemental material for Comparing Methods for Measurement Error Detection in Serial 24-h Hormonal Data Click here for additional data file. Supplemental material, Appendix_2_final_Spoel__Choi_1 for Comparing Methods for Measurement Error Detection in Serial 24-h Hormonal Data by Evie van der Spoel, Jungyeon Choi, Ferdinand Roelfsema, Saskia le Cessie, Diana van Heemst and Olaf M. Dekkers in Journal of Biological Rhythms
