# Supplementary material for: Comparing Methods for Measurement Error Detection in Serial 24-h Hormonal Data
Source: J Biol Rhythms. 2019 Jun 12;34(4):347–63. doi: 10.1177/0748730419850917 (PMC6637814; doi:10.1177/0748730419850917)
Supplement: Appendix_1_Spoel_and_Choi – Supplemental material for Comparing Methods for Measurement Error Detection in Serial 24-h Hormonal Data [file Appendix_1_Spoel_and_Choi.pdf]

Appendix 1: Details on the EM algorithm to detect outliers

For each of the hormones separately, the EM algorithm was applied to the residuals of all subjects simultaneously, where the residual of the ith measurement of subject j was calculated as $R_{ij} = Y_{ij} - \hat{Y}_{ij}$, with Y_{ij} the observed measurement and $\hat{Y}_{ij}$, the moving average smoothed estimate.

We assumed that there were two types of measurements: true measurements and erroneous measurements. We expected that the residuals of the true measurements had standard deviations close to 0, while erroneous measurements had a much larger standard deviation.

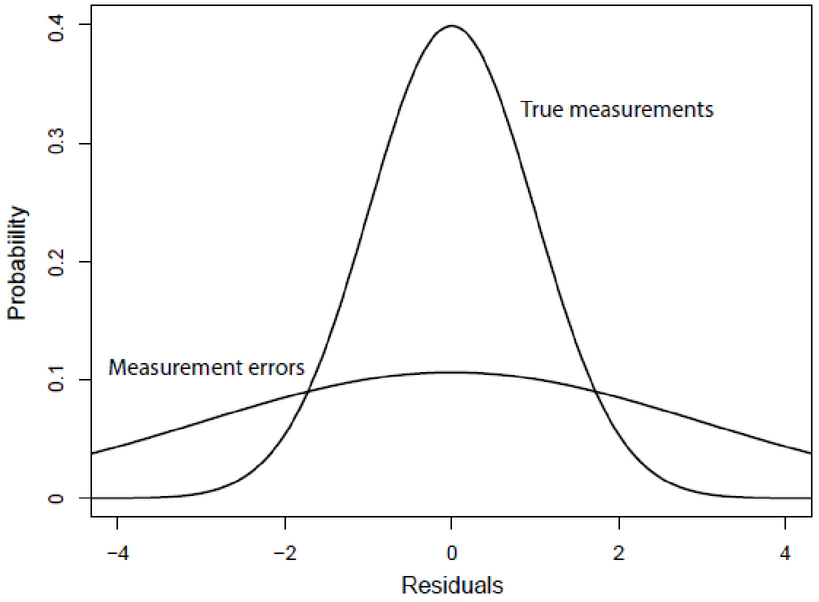

The (unobserved) indicator variable Z denotes whether a measurement is an error, with Z_{ij}=1 if the ith measurement of subject j is an error and Z_{ij} =0 if it is a true measurement. The proportion of erroneous measurements Pr(Z_{ij}=1) is denoted by π_e. We assumed that residuals R of true measurements were normally distributed with mean 0 and standard deviation σ_1 while the residuals of the erroneous measurements were normally distributed with mean 0 and standard deviation σ_2, with $\sigma_2 \gg \sigma_1$. The proportion of erroneous parameters π_e and the standard deviations σ_1 and σ_2, can be estimated using maximum likelihood. The complete likelihood of the data is

$$L(\sigma_1, \sigma_2; R, Z) = \prod_{ij} f(R_{ij};\ \sigma_1)^{(1-Z_{ij})} f(R_{ij};\ \sigma_2)^{Z_{ij}},$$

with $f(;\ \sigma_i)$, the normal density with mean 0 and standard deviation σ_i. Because the Z_{ij} are unobserved, the EM algorithm is applied, with following EM steps:

E step: given current estimates p_e s_1 and s_2 for π_e σ_1 and σ_2 , the expected probability to be an error is estimated using Bayes formula:

$$\Pr(Z_{ij}=1 \mid R_{ij}) = \frac{p_e f(R_{ij}; s_2)}{(1-p_e) f(R_{ij}; s_1) + p_e f(R_{ij}; s_2)} \quad (1)$$

M step: the likelihood function where the Z_{ij} are replaced by the expected probabilities that Z_{ij} is 1, is maximized.

The EM steps are repeated until convergence. The final estimates p_e ,s_1 and s_2 are filled in in equation (1). This yields for each measurement an estimated probability to be an error measurement.

The EM algorithm was applied in R version 3.5.1, using the normalmixEM function of the package mixtools.

Reference

Benaglia T, Chauveau D, Hunter DR, Young D (2009). mixtools: An R Package for Analyzing Finite Mixture Models. Journal of Statistical Software, 32(6), 1-29. URL http://www.jstatsoft.org/v32/i06/.
