# Supplementary material for: Comparing Methods for Measurement Error Detection in Serial 24-h Hormonal Data
Source: J Biol Rhythms. 2019 Jun 12;34(4):347–63. doi: 10.1177/0748730419850917 (PMC6637814; doi:10.1177/0748730419850917)
Supplement: Appendix_2_final_Spoel__Choi_1 – Supplemental material for Comparing Methods for Measurement Error Detection in Serial 24-h Hormonal Data [file Appendix_2_final_Spoel__Choi_1.pdf]

## Appendix 2: Detecting outliers in 24-hour hormonal data: a simulation study

### 1. Data generation

We simulate measurements for 5 hormones; glucose, insulin, thyroid stimulating hormone (TSH), cortisol and growth hormone (GH), according to their physiological characteristics and the laboratory setting where our sample was drawn. This setting was reproduced in simulation as described below:

- 24 hours with measurements every 10 minutes, in total 144 measurements per hormone and person.
- 3 meals at time 0, 18, 54.
- Night from time 84 to time 138.

For each hormone we generated measurements. The mean hormone value at time t, Y(t) consisted of a constant baseline level and one or more peaks using an absorption/elimination model. A peak starting at $t_s$ has the form:

$$Y(t) = C_0 + C_1 (t>t_s)(e^{-\lambda_e t}-e^{-\lambda_a t}),$$

where $C_0$ determines the minimum hormone values over time, $C_1$ the peak value and $\lambda_a$ and $\lambda_e$ the rate of absorption and elimination of the hormone in blood. The latter is directly related to the half-life of the hormone by $\lambda_e$=ln(2)/half-life. Random between and within person variation was added to the generated mean values. The specific minimum and location and duration of peaks, and the random intra and inter person variation were based on the observed patterns in our data. Specific features of each hormone are:

- Glucose: Three clear peaks after meals where the third one is slightly higher than others. At night, the hormone level is stable and low, and the variation is smaller. Physiologically, glucose levels cannot be below 2.8 mmol/L.
- Insulin: Three clear peaks after meal, and the hormone is highly correlated with glucose (corr.=0.75). At night, the hormone level is stable and low and the variation is smaller.
- TSH: One big peak, where the hormone builds up in the evening from 6 pm (t=54) with highest levels at 11 pm (t=84), with large variation.
- Cortisol: Peaks at the end of the night.
- GH : Sharp peaks, and the number of peaks varies from 0 to 20 across the individuals.

Inter-person variation is generated by varying the highest concentration reached during peaks, following a normal distribution (specific parameters are provided in the table below). For TSH, cortisol and GH, the location of the peaks also varies across the people. In this way we generated 24-hour hormonal data for 38 simulated subjects. Table A1 shows the specific parameters used for simulating 24-hour hormonal data of 38 individuals.

**Table A1 Parameters for generating 24-hour glucose, insulin, TSH, cortisol and GH data**

| | Glucose [mmol/L] | Insulin [mU/L] | TSH [mU/L] | Cortisol [µmol/L] | GH [mU/L] |
|---|---|---|---|---|---|
| Starting value ($C_0$) | 3.8 | 6.6 | 1 | 0.05 | 1 |
| Number and location of peaks | 3 peaks, increase starts at mealtimes | 3 peaks, increase starts at mealtimes | One wide peak, increase starts between t=45 and 65 | 3 peaks, Increase starts between (i) t=75 and 100, (ii) between t=100 and 124, and (iii) between 124 and 140 | 0 to 20 peaks, increase starts from t=0 and 143 |
| Half-life | 35 min | 35 min | 120 min | 50 min | 10 min |
| Intra-person variation (SD) | Day 0.50, Night 0.25 | Day 6.5 Night 3.2 | 0.17 | 0.03 | 0.27 |
| Mean and SD of peaks: first peak (i), second peak (ii), third peak (iii), with inter person Sd | (i) 4 (0.5), (ii) 4 (0.5), (iii) 7 (0.7) | 90 (5) | 2.5 (0.5) | (i) 0.3 (0.1), (ii) 0.4 (0.1), (iii) 0.5 (0.1) | 15 (1) |
| Remarks | Values <2.8 are set to 2.8 | Values <2.8 are set to 2.8 | Values <1 are set to 1 | Values <0.05 are set to 0.05 | Values <0.2 are set to 0.2 |
| Absorption/elimination rate | $\lambda_a = 1.1 \lambda_e$ | $\lambda_a = 1.1 \lambda_e$ | $\lambda_a = 1.1 \lambda_e$ | $\lambda_a = 2 \lambda_e$ | $\lambda_a = 1.1 \lambda_e$ |
| Comments | | Log transformation | | | Log transformation |

In each individual, for each hormone we generated measurement errors at 14 time points. To generate random measurement errors in each hormone at 7 randomly selected time points (5% out of 144 points), we replaced the true measurement by an error measurement drawn from a uniform distribution with a wide range (-10 x intra-person SD to 15 x intra-person SD). Furthermore we generated related dilution errors at 7 time points which were the same across all hormones for one individual.  The dilution errors were generated by dividing the original measurement by 2.

## 2. Simulation results

Figure A1 shows simulated 24-hour hormonal data for glucose, insulin, TSH, cortisol and GH of the first two generated individuals are shown. The hormone specific measurement errors are indicated by a red dot. The dilution errors are indicated by a green dot.

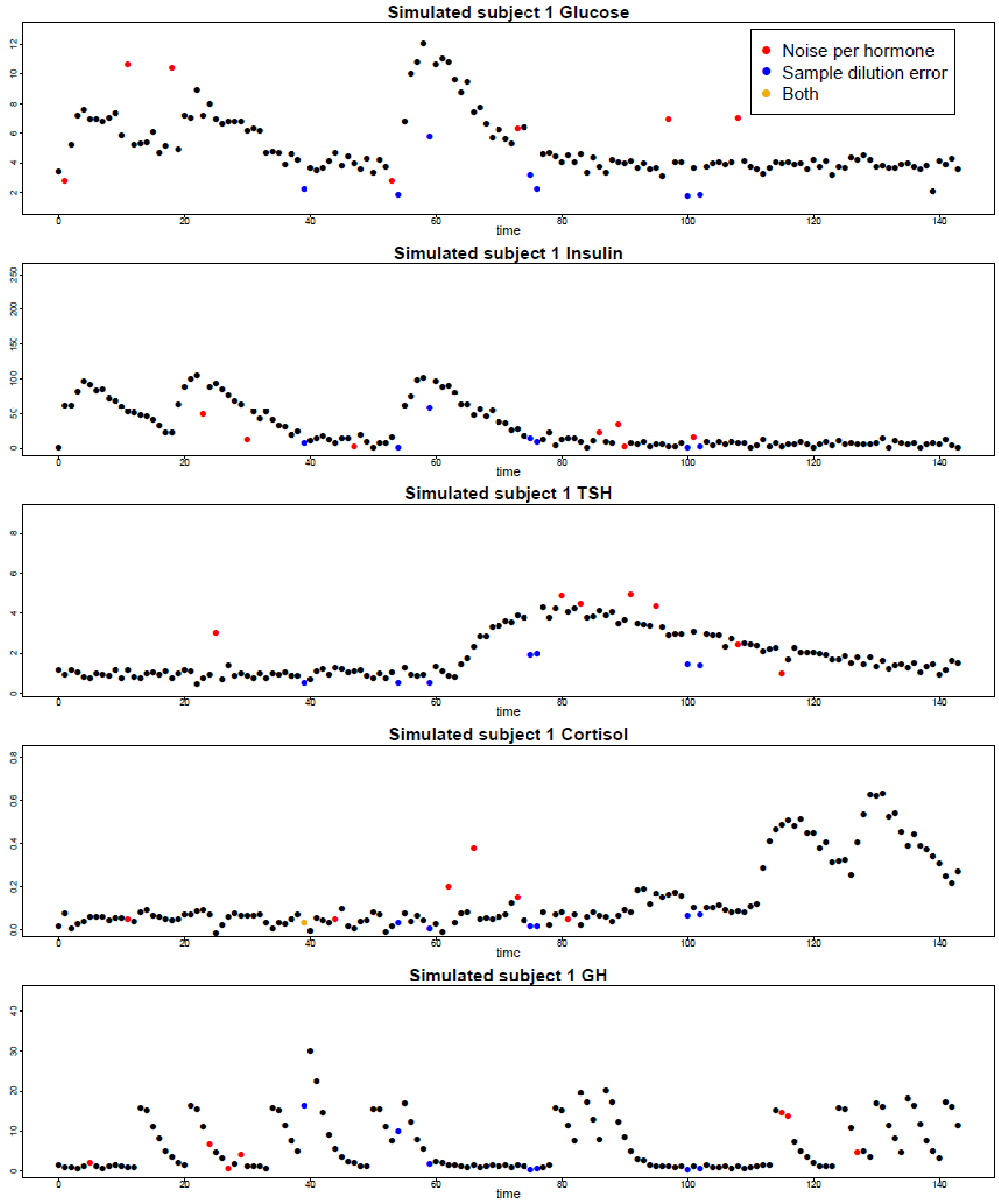

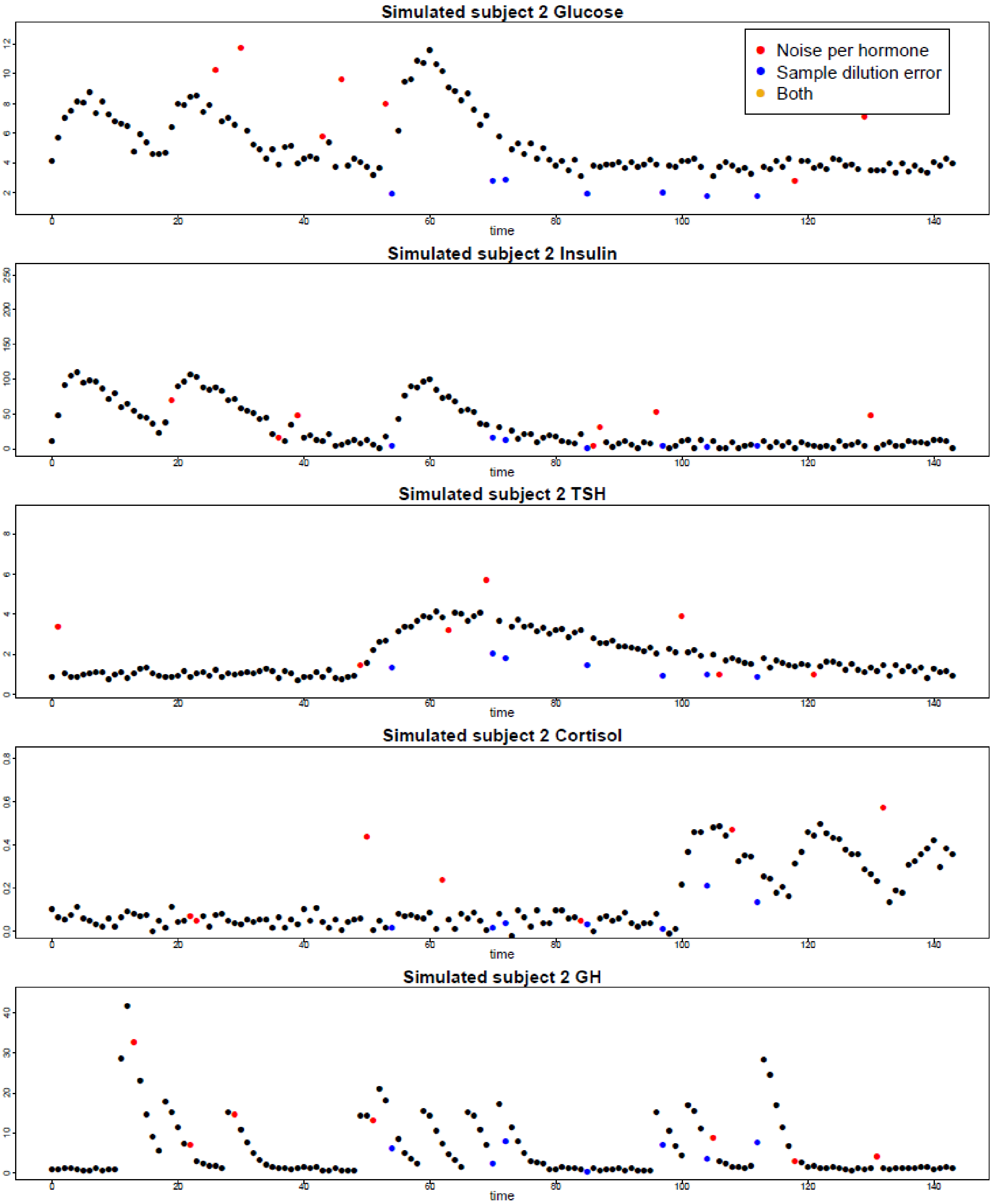


**Figure A1 Simulated 24-hour glucose, insulin, TSH, cortisol, and GH data of the first two generated individuals**

Figure A2 displays how many points are indicated as measurement errors by each method averaged across the 38 simulated subjects. The EM algorithm indicated the highest number of measurement errors, followed by the stepwise approach. Especially for the hormones where the intra-person variation was larger during day than during night (glucose and insulin), the EM algorithm indicated high numbers of measurement errors.

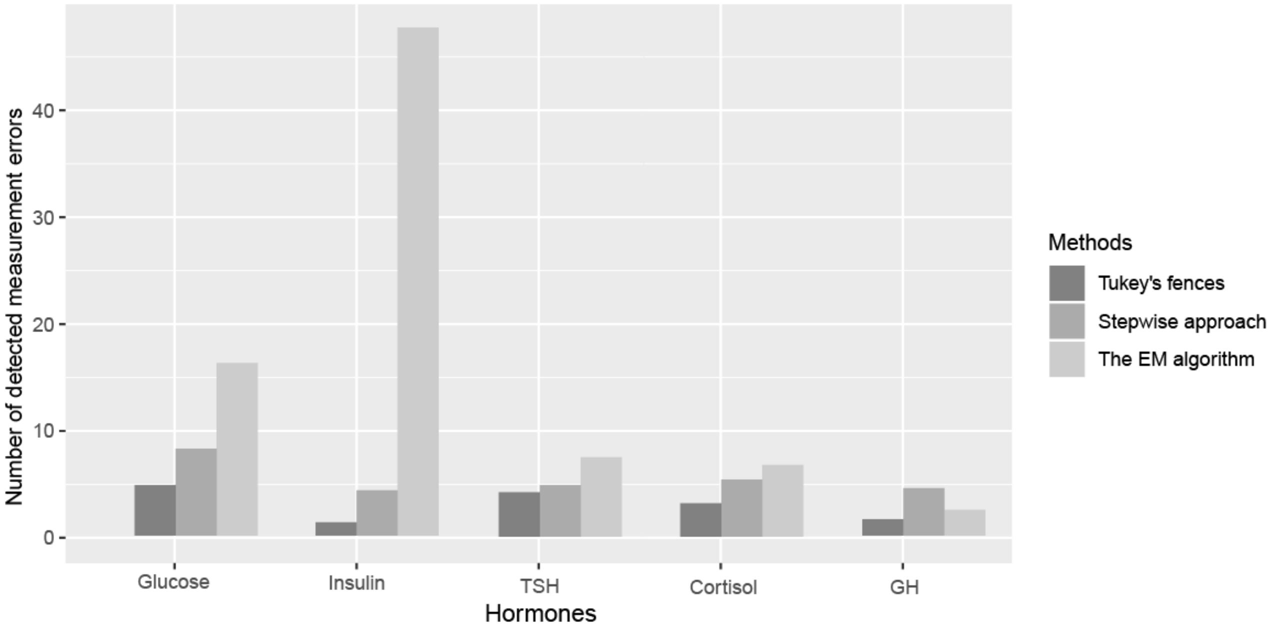


**Figure A2 Simulated 24-hour glucose, insulin, TSH, cortisol, and GH data of the first two generated individuals**

**Table A2 Percentage of true errors detected and true measurement wrongly indicated as error by each method stratified by random error and dilution error.**

| | | Random error | | Dilution error | |
|---|---|---|---|---|---|
| | | True errors detected (%) | True measurements wrongly indicated as error (%) | True errors detected (%) | True measurements wrongly indicated as error (%) |
| Stepwise approach | Glucose | 22.18 | 4.80 | 92.86 | 1.19 |
| | Insulin | 4.51 | 2.86 | 49.25 | 0.58 |
| | TSH | 18.42 | 2.59 | 53.76 | 0.79 |
| | Cortisol | 24.06 | 2.67 | 49.62 | 1.36 |
| | GH | 8.27 | 2.82 | 49.25 | 0.73 |
| | mean | 15.49 | 3.15 | 58.95 | 0.93 |
| Tukey's fences | Glucose | 34.96 | 1.67 | 29.32 | 1.96 |
| | Insulin | 7.89 | 0.50 | 4.14 | 0.69 |
| | TSH | 31.58 | 1.42 | 27.82 | 1.61 |
| | Cortisol | 31.95 | 0.65 | 7.52 | 1.90 |
| | GH | 7.89 | 0.71 | 2.63 | 0.98 |
| | mean | 22.86 | 0.99 | 14.29 | 1.43 |
| The EM algorithm | Glucose | 60.53 | 8.70 | 90.98 | 7.15 |
| | Insulin | 74.81 | 30.89 | 77.82 | 30.73 |
| | TSH | 49.25 | 2.92 | 46.24 | 3.07 |
| | Cortisol | 43.98 | 2.65 | 18.80 | 3.94 |
| | GH | 8.65 | 1.31 | 4.14 | 1.54 |
| | mean | 47.44 | 9.29 | 47.59 | 9.29 |

Table A2 shows what percentage of true errors (random errors and dilution errors) were detected by each method and how much non-errors were identified as errors by each method. When it comes to detecting true error, the EM algorithm performed best. However, the EM algorithm also indicated the most non-errors as measurement errors. Especially for insulin, the number of  true measurements which were indicated as error was extremely high. This is explained by the fact that the intra person variation in insulin differed between day and night, and the insulin residuals were not normally distributed without log transformation. The percentage of non-error detected as measurement error was much lower in Stepwise approach and Tukey's fences than in the EM algorithm. Stepwise approach is to be preferred when detecting dilution errors.
